# Ukrainians under continuous traumatic stress: towards increasing the diagnostic condition’s clinical utility

**DOI:** 10.3389/fpsyt.2026.1872305

**Published:** 2026-09-10

**Authors:** Andreas Maercker, Lukas Steinkämper, Brigitte Lueger-Schuster

**Affiliations:** 1 Department of Psychology, University of Zurich, Zürich, Switzerland; 2 Faculty of Psychology, University of Vienna, Vienna, Austria

**Keywords:** clinical utility, continuous traumatic stress, diagnostic conditions, refugees, Ukrainian population

## Abstract

**Background:**

The World Health Organization would include a diagnosis of Continuous Traumatic Stress (ConTS) Reaction or Disorder in its ICD-11 classification system if there were more empirical evidence to support it. However, research on people experiencing ongoing traumatic experiences is always difficult from an ethical standpoint. Our modest study re-examined the structural validity of the Goral et al. CTS (2021) questionnaire and some predictive relationships in samples of Ukrainians in their home country compared to externally displaced Ukrainians (to Austria).

**Method:**

N = 46 Ukrainians in their war-threatened homeland (mean age = 43 years) and N = 64 Ukrainian refugees in Austria (mean age = 37 years) were examined via online data collection. In addition to the ConTSR scale (ConTSR), traumatic life events (LEC-5), PTSD and CPTSD, and other major psychopathologies were examined. The structural validity of the ConTS was calculated using internal reliabilities of existing factor analyses (FA) and our own exploratory FA.

**Results:**

The factorial structure suggests a two-factor structure with the factors: exhaustion/rage and fear/betrayal. The theoretically more meaningful three-factor structure only achieves borderline reliabilities. Those who stay in Ukraine show higher values in the ConTS subscale exhaustion/rage (and in the single item *sense of danger*) compared to the values of those who escaped to Austria. The highest correlations were found between the ConTS total scale and the CPTSD cluster of disturbances in self-organization (*r* = .70) and depression (*r* = .66), while there were lower correlations with classic PTSD (*r* = .53) and general emotion dysregulation (*r* = .55).

**Outlook:**

This study with two meaningful subsamples provides new insights into the factor structure of the internationally most widely adopted ConTS concept. It indicates for the ConTS concept that it is not classic PTSD but rather CPTSD that shows conceptual similarities.

## Introduction

As part of its responsibility for revising the ICD-11, the WHO Department of Mental Health and Substance Use has, in principle, signaled a positive stance toward including a Continuous Traumatic Stress (ConTS)^1^ Disorder or reaction in the diagnostic catalog (2). In particular, two members of the commission, Ashraf Kagee from South Africa and Daya J. Somarasundaram from Sri Lanka, had called for this new additional diagnostic category because there are too many individuals who are not living in a post-traumatic situation but are in the midst of one—for example, amid extremely high levels of poverty-related violence in South Africa or amid the decades-long civil war in Sri Lanka. At the time (around 2015), however, the WHO decided that it was still too early for an official diagnosis because there was insufficient empirical research on the topic.

Until the mid-2010s, there was still no generally agreed-upon conceptualization of ConTS. Qualitative and quantitative studies agreed, however, that there should be a distinction from post-traumatic stress disorder (PTSD). In particular, anxiety or fear symptoms were considered clinically more significant than in PTSD (cf. 3). However, it remains a goal for psychopathology to achieve a consensus definition that could be applied across multiple studies if a proposed diagnosis should have a chance to be included in the ICD-11 (4).

The chapter “Mental, behavioral, or neurodevelopmental disorders” of the ICD-11 (5) was revised with the methodological requirement that all diagnostic definitions should be characterized by high clinical utility and cultural applicability (6). Crucially, these two requirements were met by ensuring that the list of features or criteria for the diagnostic definitions was not too extensive. Thus, for the PTSD definition, which contains 21 criteria in the DSM-5, only six criteria (referred to there as “features”) were specified in the ICD-11. Nevertheless, clinical agreement for the PTSD diagnosis was roughly the same in the ICD-11 as in the DSM-5, despite the latter significantly expanded the list of symptom criteria (7, 8).

In 2021, Goral and colleagues proposed a ConTS concept that distinguishes three symptom groups: exhaustion/detachment, rage/betrayal, and fear/helplessness. For many psychiatrists and psychologists familiar with the issue of ongoing traumatization in war and crisis zones, these three symptom groups made a great deal of sense from a clinical perspective (see 2). People suffering from the stresses of ongoing wars, civil wars, gang violence, are likely to show a wide range of physical and quasi-neurological (exhaustion/detachment), anxious-desperate (fear/helplessness), and accusatory-bitter (rage/betrayal) symptoms. From 2021 to the present (April 2026), a total of about fifty scientific papers have referenced this concept, whilst only under ten of these studies have applied it.^2^ Data collection and studies conducted under ongoing war or extreme crisis conditions pose an ethical problem: those affected actually need help with their existential problems and have often no time to participate in research projects. However, the online data collection method makes this aspect somewhat easier, resulting in a wealth of studies involving people in current wars (e.g. 9).

The factor structure of the original ConTS questionnaire (10) was not confirmed in a state-of-the-art Ukrainian translation with cultural item adaptation (11). For this study, 619 adolescents and young adults from an academic institution (students and faculty) who lived in a district (oblast) near the border with Belarus were surveyed. Although they lived under the constant direct threat of Russia invading their district via Belarus during the first two years of the war, there was almost no Russian bombing in this area because it was very far from Russia’s direct border. The study resulted in a two-factor solution, with the factors named “Exhaustion and rage” and “Fear and betrayal.”

The dire situation for the Ukrainian population caused by Russia’s full-scale invasion since February 22, 2022, has led to several million refugees leaving the country (12). The majority of them live in other European countries, while a nearly equal number are internally displaced persons. In the case of Ukraine, another relevant distinction applies: there are displaced persons from the Russian-occupied territories in the East part of the country; some of them have been displaced from these areas since the first phase of Russia’s war of aggression in 2014.

Based on these initial conditions, the following research questions arise for the present study on the still very new ConTS concept. How does the traumatic stress experienced by those who emigrated due to the war compare to that of those who remained in Ukraine? Are the three factors in the original Israeli sample more plausible than the two from the Ukrainian study? Is ConTS associated only with the existence of current war and other severe acts of violence, or are ConTS symptoms also present in people who have fled war and crisis zones? How are the associations between the ConTS symptom groups and other measures of psychopathology?

## Methods

### Sample

N=46 Ukrainians in their war-threatened homeland and N = 64 Ukrainian refugees in Austria were examined via online data recruitment. In total, 11 organizations distributed the study to eligible participants in Austria and Ukraine. Participants were recruited via flyer and postings in psychological counseling services for emigrated Ukrainian civilians in Austria, and by Ukrainian researchers in Ukraine. The study employed opportunistic sampling methods due to restricted resources and difficulty of recruitment in war-threatened areas. In Ukraine, participants were recruited from six different geographical regions: East part of Ukraine, West part of Ukraine, Kyiv, Northern Ukraine, Central Ukraine, and Southern Ukraine. Data collection was conducted from 7th of May until 30th of July 2025. Participants’ sociodemographic characteristics differentiated by the place of residence are displayed in **Table 1**. For confidentiality reasons, the length of stay of migrants in Austria was not recorded. Included in the study were civilians with Ukrainian nationality, at least 18 years of age, and with current residence either in Ukraine or Austria. Individuals with a current diagnosis or history of a mental disorder, including psychiatric hospitalization, and former or current military affiliation were excluded (the latter because they pertain to the field of professional trauma). The pre-analysis plan (13) was preregistered on the Open Science Framework (14).

**Table 1:** Sociodemographic characteristics and summarized traumatic life events of the two participant subgroups.

Variable	In Austria (n = 64)		In Ukraine (n = 46)		Test statistic	Effect size
	*M*	*SD*	*M*	*SD*	*t*(108)	Cohen’s *d*
Age	36.9	10.60	43.4	12.01	-2.99**	.58
	*n*	%	*n*	%	*x*^2^(1)	Cramér’s *V*
Gender					0.44	.06
Female	57	89.1	39	84.8		
Male	7	10.9	7	15.2		
Education level (highest)					7.46**	.26
School or high school	15	23.4	2	4.3		
Bachelor, Master, PhD	49	76.6	44	95.7		
Displacement					2.49	.15
Not displaced	3	4.7	6	13.0		
Displaced	61	95.3	40	87.0		
Traumatic life events (sum score [0-17])						
War-related	3.1	1.37	2.6	1.71	1.66	.32
Civilian (other events)	4.1	2.21	2.4	2.14	3.82***	.74

**p <.01. ***p <.001.

The participants who remained in Ukraine were statistically older (*M* = 43 years) than those living in Ukraine (*M* = 37 years). In addition, those living in Ukraine had even higher educational attainment (96% held university degrees) than the immigrants to Austria, who were still relatively highly educated (77% held university degrees).

### Measurements

Continuous Traumatic Stress Response (ConTSR): The scale developed by Goral et al. (10) comprises eleven items using a 4-point scale ranging from 0 (Not at all) to 3 (Severe). It was translated into Ukrainian by Zasiekina et al. (11). In the process, semantic meanings were slightly adjusted for cultural reasons, e.g., in item 8 from “horror” to “unrest” (*тривогу*) and item 9 from rage to tantrum (люті).

Life Events Checklist (LEC-5): To measure exposure to traumatic life events, this 17-item LEC-5 scale was used (15). The present study scored responses to scale points 1 - 4 (i.e., happened to me, witnessed it, learned about it, part of my job) as traumatic exposure, which is in accordance with the DSM-5 criterion A of post-traumatic stress disorders (PTSD). The LEC-5 encompasses traumatic life events related to acts of war—from these, the following subscales were formed *ad hoc* for this study: “war-related” (sum of the seven items: Assault with a weapon; Combat or exposure to a war zone; Captivity; Fire or explosion; Sudden violent death; Serious injury, harm, or death caused to someone else; Severe human suffering [0–7]) and “Civilian (other events)” (all other ten items from LEC’s appendix [0–10]).

International Trauma Questionnaire (ITQ): This questionnaire by Cloitre et al. (16) measures the symptoms of post-traumatic stress disorder (PTSD) and complex PTSD (CPTSD) in two parts; the latter adds the symptoms of Disturbances in Self-Organization (DSO), which constitute CPTSD, to the PTSD symptoms. As a result, the ITQ yields two scores: one for PTSD and one for DSO. The ITQ items are rated on a scale from 0 (Not at all) to 4 (Extremely).

International Depression Questionnaire (IDQ): This new questionnaire by Shevlin et al. (17) captures the ICD-11 symptoms typical of a depressive episode. The ten items have a response format ranging from 0 (Never) to 4 (Every Day).

International Anxiety Questionnaire (IAQ): This questionnaire by Shevlin et al. (17) captures the ICD-11 version of anxiety symptoms typical for Generalized Anxiety Disorder. The nine items have a response format ranging from 0 (Never) to 4 (Every Day). The Ukrainian-language versions of the LEC-5, ITQ, IDQ, and IAQ were sourced from the ITC (18).

Difficulties in Emotion Regulation Scale (DERS): This questionnaire by Gratz and Roemer (19) measures emotional dysregulation, which is described in terms of difficulties in emotion regulation across 6 factors (i.e., acceptance, goal-directed behavior, impulse control, emotional awareness, emotion regulation strategies, emotional clarity). The 36 items use a response scale ranging from 1 (Almost never) to 5 (Almost always). The Ukrainian translation was developed by Sak and Fedotova (20).

### Data analytic procedure

For the subgroup comparisons, t-tests and mainly Cohen’s *d* effect sizes were calculated. Due to the small total sample size, only an exploratory factor analysis (Principal Axis Factoring: PAF) was conducted to assess the factor structure, rather than a confirmatory one. The PAF was conducted on the 11 items of the ConTSR using Kaiser normalization and oblimin rotation, as correlations among components were expected. To validate the factor results, the potential factors were examined for internal consistency (Cronbach’s *α*). First-order correlations were calculated for convergent and discriminant validation. Participants with missing data were excluded from statistical analyses.

### Ethics approval

The study was approved by the Ethics Committee of the University of Vienna.

## Results

The analysis of traumatic life events in both subgroups ([civilian emigrants] in Austria; civilians in Ukraine) revealed that, statistically, there is no difference between the two groups in the number of “war-related” traumatic events experienced (see **Table 1**, bottom rows). When considering effect sizes, there is a slightly higher number of “war-related” traumatic events in the subgroup of individuals who emigrated to Austria, with a small effect (Cramer’s *V* = 0.32). For “civilian/other” life events, there is a significant difference, with more such events among those who emigrated to Austria (Cramer’s *V* = 0.74).

To ensure that the two subgroups could be combined into one sample for the subsequent assessment of the factor structure, group differences were examined for all individual items (**Table 2**).

**Table 2:** Descriptive statistics and mean comparison of Ukrainian ConTSR scale in two subsamples.

Item or subscale	In Austria (n = 64)		In Ukraine (n = 46)		t(108)	Cohen’s d
	*M*	*SD*	*M*	*SD*		[CI 95%]
1. Feeling unmotivated	2.25	0.67	2.15	0.76	0.72	.14 [-.24,.52]
2. Feeling mentally exhausted	2.31	0.77	2.04	0.92	1.66	.32 [-.06,.70]
3. Feeling a sense of danger	1.81	0.75	2.35	0.77	-3.65***	.71 [-1.09, -.31]
4. Feeling no meaning	2.11	0.96	1.83	0.88	1.58	.31 [-.08,.69]
5. Difficulty controlling emotions	2.00	0.59	1.80	0.65	1.64	.32 [-.07,.70]
6. Difficulty trusting people	1.97	0.71	1.67	0.60	2.29*	.44 [.06,.82]
7. Feeling misunderstood	2.03	0.93	1.65	0.67	2.36*	.46 [.07,.84]
8. Feeling fear or horror	2.36	0.80	2.28	0.69	0.52	.10 [-.28,.48]
9. Episodes of rage	1.89	0.76	1.74	0.65	1.10	.21 [-.17,.59]
10. Feeling betrayed	1.92	0.90	1.63	0.85	1.72	.33 [-.05,.71]
11. Feeling inability to protect	2.30	0.94	2.09	0.89	1.18	.23 [-.15,.61]
ConTSR total	22.95	5.17	21.24	5.37	1.70	.33 [-.06,.71]
ConTSR Exhaustion/Rage	12.59	3.02	11.22	3.57	2.18*	.42 [.04,.80]
ConTSR Fear/Betrayal	10.36	2.72	10.02	2.54	0.66	.13 [-.25,.51]

ConTSR, Continuous Traumatic Stress Response. *p* values were adjusted for multiple comparisons using the Benjamini-Hochberg correction.

*p <.05. ***p <.001.

This subgroup comparison revealed that, out of eleven items, only three showed significant differences in effect size. The item “Feeling a sense of danger” was stronger in the “In Ukraine” subgroup (effect size *d* = 0.71). Two items were stronger in the “In Austria” subgroup: “Difficulty trusting people” (*d* = 0.44) and “Feeling misunderstood” (*d* = 0.46).

For the PAF, the Kaiser-Meyer-Olkin (KMO) measure indicated that the data were suitable for factor analysis (KMO = .80) and the Bartlett’s test of sphericity was significant, *x*^2^(55) = 369.92, *p* <.001. Two components with eigenvalues above 1 emerged. A third component only reached a borderline value of.985. The PAF supports a two-factor solution which explained a total of 40.6% of variance in ConTS values. Factor 1 explained 32.5% and factor 2 explained 8.1% of this variance, respectively. Items 3, 8, 10, 11 clustered on factor 1 (i.e., fear/betrayal). Items 1, 2, 4, 5, 6, 7, 9 clustered on factor 2 (i.e., exhaustion/rage). In this sample, item 6 (“difficulty trusting people”) loaded onto exhaustion/rage, instead of fear/betrayal of the two-factor solution.

The two-factor structure (11) was corroborated rather than the three-factor structure (10) of the CTSR regarding internal consistencies (**Table 3**). The two-factor structure achieved an average Cronbach’s alpha of 0.73. The three-factor structure achieved an average Cronbach’s alpha of 0.67. Thus, the two-factor structure tends to be preferred.

**Table 3:** Psychometric properties of Ukrainian ConTSR scale and subscales in the combined sample.

Scale	Items	M	SD	Cronbach’s *α*
ConTSR total	11	22.2	5.3	.83
Two-factor structure				
Exhaustion/Rage	6	12.0	3.3	.80
Fear/Betrayal	5	10.2	2.6	.66
Three-factor structure				
Exhaustion/Detachment	5	10.1	2.9	.78
Rage/Betrayal	3	5.6	1.7	.61
Fear/Helplessness	3	6.6	1.9	.64

*n* = 110. ConTSR, Continuous Traumatic Stress Response.

To assess validity, a comparison of the ConTS with other psychopathologies revealed that the ConTS score showed the strongest correlation with depression (measured here as the total score for depressive symptoms) (*r* = 0.70); even higher than that with PTSD (*r* = 0.53) and the CPTSD sub-symptom DSO (*r* = 0.66) (**Table 4**).

**Table 4:** Intercorrelations of study variables in the combined sample.

Measure	1	2	3	4	5	6	7	8	9	10
1. ConTSR total	–									
2. ConTSR ER	.91***	–								
3. ConTSR FB	.86***	.58***	–							
4. ITQ PTSD	.53***	.43***	.52***	–						
5. ITQ DSO (CPTSD)	.66***	.68***	.47***	.51***	–					
6. IDQ (depr. epis.)	.70***	.67***	.57***	.51***	.75***	–				
7. IAQ (GAD)	.65***	.61***	.53***	.56***	.62***	.84***	–			
8. DERS (emot. reg.)	.55***	.54***	.42***	.39***	.58***	.52***	.51***	–		
9. LEC-5 war-related	.27**	.20*	.30**	.31**	.24*	.33***	.34***	.11	–	
10. LEC-5 civilian	.32***	.29**	.28**	.30**	.40***	.25**	.23*	.26**	.55***	–

*n* = 110. ConTSR, Continuous Traumatic Stress Response; ER, Exhaustion and Rage; FB, Fear and Betrayal; ITQ, International Trauma Questionnaire; IDQ, International Depression Questionnaire—measures depressive episodes; IAQ, International Anxiety Questionnaire—measures General Anxiety Disorder; DERS, Difficulties in Emotion Regulation Scale; LEC-5, Life Events Checklist for DSM-5.

*p <.05. **p <.01. ***p <.001.

For the ConTS Exhaustion and Rage subscale, the correlations with depressive symptoms and with the DSO component of CPTSD are nearly equal (*r* = 0.68 and 0.67). For the ConTS Fear and Betrayal subscale as well, correlations with depressive symptoms are comparatively the highest (*r* = .57), followed by equally high correlations with symptoms of generalized anxiety disorder and PTSD (*r* = 0.53 and *r* = 0.52). Difficulties in Emotion Regulation were moderately correlated with the ConTS total score and the ConTS Exhaustion and Rage Score (*r* = 0.55 and *r* = 0.54).

Weak correlations were found between the ConTS total score and the two subscores of the LEC-5 (the highest r = 0.28 - 0.32).

## Discussion

With two subsamples of Ukrainians currently affected by the war—for ethical and practical reasons difficult to recruit—several aspects of the ConTS concept were examined based on the framework proposed by Goral et al. (10). The primary objective of these analyses was to determine whether this clinically useful concept could support the introduction of a diagnostic category for Continuous Traumatic Stress Reaction in a revision of the ICD-11 (2).

The first research question was how to characterize the sample—or more precisely, the two subsamples. The subsamples were small to medium in size. As in most studies, women were more likely to participate than men (rounded: 85% versus 15%). Educational attainment was very high: approximately 85% held university degrees. The assessment of traumatic stressors revealed that the vast majority of both subsamples (just over 90%) reported having been forcibly displaced by the war. The list of traumatic life events revealed no difference between the subsamples in the frequency of war-related life events, but a difference in the prevalence of civilian traumas in the group that emigrated to Austria. A *post-hoc* hypothesis for this difference is that those who were particularly psychologically traumatized were more likely to go abroad because they feared that the war-related traumatic exposures would place a particularly heavy burden on their lives.

The second research question was whether the three-factor (10) or two-factor solution (11) of the ConTS is plausible for the (combined) sample. Goral et al. (10) had previously examined an Israeli sample that was more representative in terms of certain demographic characteristics than the samples in the present study: in addition to approximately 65% with academic education, 35% had no academic degrees. Income—not recorded in the present study—was below the national average for approximately 70% of the sample; finally, there were also approximately 35% men, which is more than in the present study. The study with Ukrainian participants (11)), led to a two-factor solution, had younger participants on average (mean 19 years), and it also consisted predominantly of women (87%), and all were university students or staff.

We also found a two-factor solution in an analysis appropriate for the comparatively small sample size (exploratory factor analysis and internal consistency calculations of the factors). This maps the clinical phenomena of exhaustion and rage onto one factor, and fear and betrayal onto the second factor. Clinically, these factors are somewhat more difficult to interpret than the three-factor solution proposed by Goral et al. (10): Exhaustion/Detachment (lack of energy), Rage/Betrayal (interpersonal affects), Fear/Helplessness (existential threat). It could be that the EFA results of our study primarily reflect frequency patterns (21), accordingly, exhaustion and rage would be clinically more frequently present in the sample than fear and betrayal.

Due to the specific characteristics of our sample (many more women, higher education), these factors may also have contributed to the differences in the factor solutions. Therefore, it is left to future studies with larger and demographically more balanced samples to make more comprehensive statements about the optimal ConTS factor structure. In any case, the high internal consistencies of the present study indicate that ConTS symptoms represent a coherent clinical picture.

The third research question focused on differences in the items and subscales of the ConTSR scale. Here, only one of eleven items was significantly elevated, whilst 3 of 11 items showed significant small-to-medium effects. Consistent with the nature of the samples, this “feeling a sense of danger” was higher among those who stayed in Ukraine. Conversely, the Exhaustion/Rage subscale was higher among those residing in Austria as immigrants. One interpretation of this could be that they were no longer in a state of direct threat due to acts of war. Therefore, “secondary war” emotions—in which exhaustion resembles mental defeat and rage resembles anger—may have been higher among them (22, 23).

The final research question concerned the positioning of the ConTSR scale in psychometric space. In summary (regarding the total ConTSR score), the strongest associations were found, on the one hand, with depressive symptoms and, on the other hand, with the three “disturbances in self-organization” (DSO), the key symptom clusters of complex PTSD. Clinical anxiety symptoms followed closely. This is not surprising, as depressive and anxiety symptoms are often transdiagnostic indicators of generally poor mental well-being (24). Further studies with Ukrainians living under wartime conditions have shown that DSO symptoms are highly correlated with those of the ConTSR (25, 26). Accordingly, people affected by war feel impaired in the DSO domains of affect regulation, negative self-concept, and relationship quality, although the cross-sectional study design does not allow for determining how long these impairments persist.

Regarding the establishment of clinical ConTSR—as mentioned earlier as a potential diagnosis in the ICD or DSM systems—it should be noted that the correlation between the ConTSR total score and the “classical” PTSD total score, at *r* = 0.53, extends the levels of the three previously mentioned psychological measures. Thus, the psychological disorder domains of acute, persistent traumatization and post-traumatic stress disorder seem sufficiently distinct from one another and measure independent phenomena. It should be noted once again here that both entities are, in and of themselves, related to traumatic stress. However, those affected by more severe trauma, in particular, do not feel represented by the classic triad of PTSD symptoms (i.e., intrusion, avoidance, and hyperarousal). This result, too, is a “message” to the fields of psychopathology that continue to investigate the disorder category of continuous traumatic stress disorders (e.g., 1, 2). It should be briefly noted that general emotional dysregulation, as measured by the Difficulties in Emotion Regulation Scale, also shows a comparatively lower overlap with the much more specifically defined ConTSR symptomatology. The spectrum of symptoms ranging from exhaustion to betrayal (ConTSR) is distinct from, for example, problems with impulse control or emotional awareness (as measured by the DERS).

## Limitations

This study does not claim to provide definitive insights into ConTSR; rather, it aims to be a building block on a longer journey toward better defining this important clinical problem area for people affected by ongoing wars or living under continuous exposure to violence. The entire sample was assessed using an online questionnaire. As with any new clinical-diagnostic construct, the gold standard—direct clinical interviews—will eventually need to be applied. The online data collection method likely also contributed to the fact that the overall and sub-samples were skewed in their sociodemographic representativeness: there were no older individuals, significantly more women, and more participants with higher academic education. Several subscales demonstrated only modest internal consistency which weakens some of the analyses. Further analysis should have included confounding variables such as duration of displacement, legal status, and socioeconomic conditions. As a result, some of the reported results remain uncertain. Nevertheless, it should be noted that, for ethical reasons and due to the resilience of people suffering from acute war-related effects, a sample such as the present one is already highly valuable. Regarding the analyses, confirmatory factor analysis had to be omitted, particularly for reasons of statistical power.

## Clinical implications

The main implication is that the PTSD psychopathology known for 40 years differs from ConTSR. The fact that many affected individuals state they do not need therapy for “post-traumatic stress disorder” as long as the danger persists can be partially explained by the present study. The problems faced by people living under continuous (war-related) violence are different—and this is well captured by the Con-TSR concept. A recent qualitative study conducted as part of an Israeli-Ukrainian collaboration found, through a survey of mental health professionals, that they feel psychologically protected by factors such as self-care, compassion satisfaction, and positive transgenerational messages (27). These factors and others can serve as starting points for supporting interventions for people who are currently and continuously traumatized.

## Data Availability

The raw data supporting the conclusions of this article will be made available by the authors, without undue reservation.

## Footnotes

1 The abbreviation ConTS was chosen to create a clearer distinction from complex PTSD (CPTSD) (1)

2 Google Scholar search by the authors on April 24, 2026.
